# Towards Environment-Aware Fall Risk Assessment: Classifying Walking Surface Conditions Using IMU-Based Gait Data and Deep Learning

**DOI:** 10.3390/brainsci13101428

**Published:** 2023-10-08

**Authors:** Abdulnasır Yıldız

**Affiliations:** Department of Electrical and Electronics Engineering, Dicle University, Diyarbakır 21280, Turkey; abnayil@dicle.edu.tr

**Keywords:** fall risk analysis, irregular walking surfaces, convolutional neural networks, inertial measurement units, walking surface detection

## Abstract

Fall risk assessment (FRA) helps clinicians make decisions about the best preventative measures to lower the risk of falls by identifying the different risks that are specific to an individual. With the development of wearable technologies such as inertial measurement units (IMUs), several free-living FRA methods based on fall predictors derived from IMU-based data have been introduced. The performance of such methods could be improved by increasing awareness of the individuals’ walking environment. This study aims to introduce and analyze a 25-layer convolutional neural network model for classifying nine walking surface conditions using IMU-based gait data, providing a basis for environment-aware FRAs. A database containing data collected from thirty participants who wore six IMU sensors while walking on nine surface conditions was employed. A systematic analysis was conducted to determine the effects of gait signals (acceleration, magnetic field, and rate of turn), sensor placement, and signal segment size on the method’s performance. Accuracies of 0.935 and 0.969 were achieved using a single and dual sensor, respectively, reaching an accuracy of 0.971 in the best-case scenario with optimal settings. The findings and analysis can help to develop more reliable and interpretable fall predictors, eventually leading to environment-aware FRA methods.

## 1. Introduction

Falls are the most significant cause of injury-related fatalities and hospitalizations among the elderly [1,2]. Every year, more than 33% of older adults in the United States fall, with 10% of these falls resulting in injuries that necessitate medical attention [3]. Environmental hazards, gait and balance disorders, and dizziness and vertigo are the most common causes of falls in the elderly, accounting for 61% of all falls [4]. Fall risk assessment (FRA) seeks to determine different risk factors associated with these causes for individuals. The choice and timing of measures to prevent fall events are guided by FRA. Despite major advancements in FRAs, fall prevention measures cannot completely eliminate falls. The majority of FRAs, such as timed-up-and-go and Tinetti tests, are conducted under controlled conditions, i.e., clinical settings [5]. Even though these tests can provide valuable insights about biological risk factors, they are not very successful in real-world conditions [6]. Furthermore, real-world performance of these tests can suffer from the Hawthorne effect, which is the change of behavior in a laboratory as a result of awareness of observation [7]. Finally, although environmental hazards and accidents are the most common sources of falls, in-lab tests cannot identify risk factors associated with interactions between the environment and individuals. Therefore, there is a need for new free-living FRA methods that involve evaluating an individual’s risk of falling in their natural environment while considering daily activities, behaviors, and environmental factors to address these constraints.

Recently, wearable technologies such as inertial measurement unit (IMU) sensors and smartwatches have made the recording and assessment of older adults’ activities in real-world environments possible [8]. This has facilitated the development of several free-living FRAs using fall predictors or markers extracted from IMU-based data. These markers include step, stance, swing, and total walking times, frequency measures, number of steps, missteps, and turns [9,10]. Mainly extracted from gait, these predictors have been reported to outperform in-lab FRAs at identifying individuals’ fall risks [11]. However, many of these predictors rely heavily on the detection of steps and exhibit variable fall prediction abilities in studies, suggesting potential instability in identifying people susceptible to falling [6]. Moreover, while these markers can be influenced by both biological and environmental conditions, the variations induced by these conditions and their effects on the predictive power of the markers have yet to be analyzed [12]. For instance, higher variability in markers reflecting step-to-step consistency (e.g., power spectral density, number of steps taken, and total activity duration) may indicate a higher fall risk. Still, if this variability results from walking on an irregular surface, it might signify an appropriate gait adaptation and a lower risk [13]. Therefore, recording environmental awareness at various time intervals has the potential to significantly enhance the interpretation and reliability of these predictors. This advancement could pave the way for the development of environment-aware free-living FRA methods.

Environment-aware free-living FRA methods would provide information about individuals’ surroundings in sync with other measurements. Especially at the times when fall predictors indicate a higher fall risk or a fall occurs, the interpretation of this data would enable: (1) clarifying uncertainties about the reason for variations in the predictors; (2) reducing the number of false alarms by ignoring environment-induced higher fall risks that are not followed by a fall; and (3) understanding the interactions between biological and environmental factors. This understanding can unravel new types of interventions aimed at eliminating the root causes of falls. In the best case, an environment-aware free-living FRA method would record the number of falls, fall predictors, and environmental conditions such as walking surfaces, existence of objects, and lighting conditions. It would interpret interactions between these variables to identify fall risks and suggest types of interventions to reduce or eliminate risks. To pave the way for an ideal FRA, this study aims to introduce a deep learning method to categorize nine walking surface conditions using IMU-based gait data.

There are several studies that have focused on the automated detection of walking surface conditions using IMU-based methods. Hu et al. [14] introduced a deep learning approach employing a long short-term memory (LSTM) model to detect walking surface conditions. Their study utilized data from gyroscopes, accelerometers, and magnetometers to identify flat or uneven brick surfaces. Hashmi et al. [15] utilized acceleration and angular velocity signals from two smartphones placed on participants’ chests and lower backs. They employed hand-engineered features, as well as support vector machines (SVM) and random forest (RF) classifiers to categorize six different walking surfaces. Another study by Hu et al. [16] explored the application of deep learning (DL) techniques for walking surface condition detection. Their database was generated using data from wearable sensors positioned on various body parts, and they investigated the impact of sensor location and different DL methods, including convolutional neural networks (CNN) and LSTM models. Additionally, Shah et al. [17] conducted research focusing on sensor locations, sensor counts, and data splitting strategies’ effects. They used linear discriminant analysis for feature extraction and multi-layer perceptron for classification. Their study utilized the dataset previously employed in Ref. [18], which included gait data collected from 30 participants wearing six IMUs while walking on various surfaces (grass, flat-even, stairs-up, stairs-down, slope-up, slope-down, banked-left, banked-right, cobblestone). Although these studies successfully demonstrated that IMU-based machine learning techniques can detect walking surface conditions, further comprehensive analysis is warranted. This includes examining the effects of different signals and signal combinations, conducting more extensive sensor location/count analyses, and addressing the detection speed aspect of the problem, including the segmentation of continuous gait data.

This study is built upon the dataset established by [18], which was gathered through the use of wearable sensors positioned on various body parts, including the trunk, wrists, thighs, and shanks. These wearable sensors incorporate an accelerometer, gyroscope, and magnetometer. The study introduces a deep learning approach aimed at identifying various walking surfaces based on gait time series data, which indirectly conveys information about the walking surfaces. The primary contributions of this research encompass:

- The recognition of nine distinct irregular walking conditions through the utilization of gait time series data in conjunction with a CNN model;
- An in-depth analysis of signal fusion, involving the combination of different signals such as acceleration, rate of turn, and magnetic field;
- Evaluation of single- and multi-sensor placements;
- Assessment and analysis of gait data segmentations into five different lengths: 100, 200, 300, 400, and 500 samples.

The paper is organized as follows. Section 2 elaborates on the gait dataset and provides an overview of the framework along with descriptions of the methods used in the study. Section 3 presents detailed experimental results, while Section 4 discusses these results. Finally, in Section 5, the study’s conclusion is reviewed.

## 2. Materials and Methods

**Figure 1:**
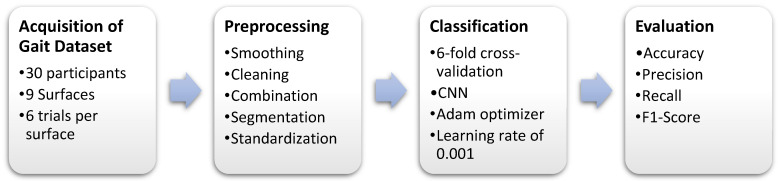
Block diagram of the proposed system.

This study proposes a deep learning framework for detecting walking surface conditions using gait signals collected from wearable sensors. Gait data from various wearable sensors were collected, preprocessed, and fused to generate inputs for the CNN. These inputs consisted of 2D matrices formed by different combinations of windowed sensor signals. The generation of inputs aimed to identify the most suitable signals (accelerometer, gyroscope), sensors, and window length. Ultimately, the performance of the proposed method was analyzed using 6-fold cross-validation. The system’s block diagram is illustrated in Figure 1.

### 2.1. Gait Database

Recently, an open gait database has become publicly available, collected by wearable sensors for human gait performance analysis on irregular and uneven surfaces [18]. The database involved 30 volunteers (15 males and 15 females), with an average age of 23.5 ± 4.3 years, height of 169.3 ± 21.5 cm, weight of 70.9 ± 13.9 kg, and body mass index of 23.7 ± 3.6 kg/m^2^. None of the participants reported any falls in the two years leading up to data collection, and none had known neurological conditions or musculoskeletal injuries that could have impacted their gait.

Participants were given instructions to perform multiple walking trials while wearing six IMU sensors (Xsens, Enschede, The Netherlands). These sensors were affixed to various body parts, including: (a) the right wrist (wrist), (b) the right thigh (thighR), (c) the left thigh (thighL), (d) the right shank (shankR), (e) the left shank (shankL), and (f) the trunk.

**Figure 2:**
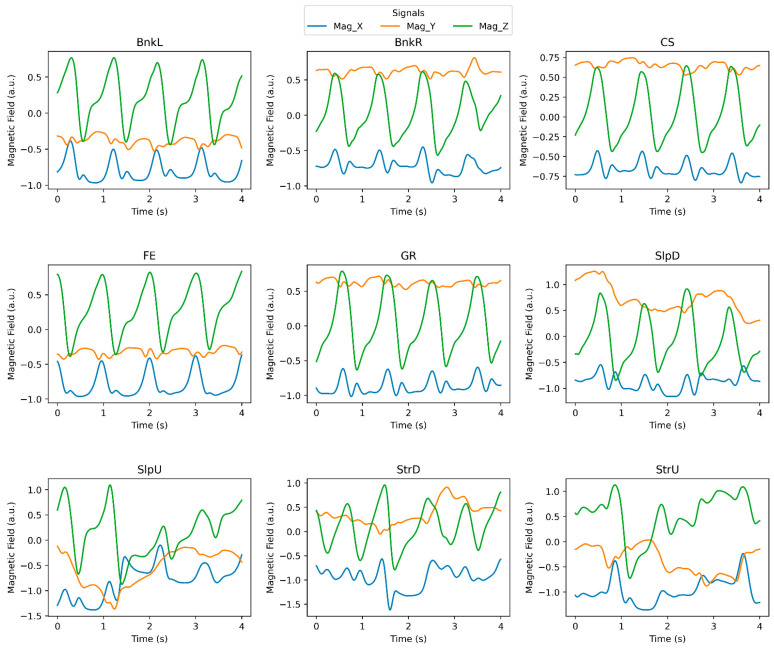
Signal patterns of 3D magnetic field recorded from the right shank sensor in various walking conditions. a.u. stands for arbitrary unit. The blue, orange, and green curves represent the magnetic field in the X, Y, and Z directions, respectively. BnkR and BnkL indicate surfaces banked to the right and left, SlpD and SlpU represent surfaces with downhill and uphill slopes, and StrD and StrU indicate descending and ascending stairs. CS, FE, and GR stand for cobblestone, flat even, and grass surfaces, respectively.

Before participants commenced their trials, the sensors underwent calibration. The trials were conducted on nine different surfaces, which were: (a) paved flat even (FE), (b) cement upstairs (StrU), (c) cement downstairs (StrD), (d) cement upslope (SlpU), (e) cement downslope (SlpD), (f) grass (GR), (g) paved surface banked left (BnkL), (h) paved surface banked right (BnkR), and (i) uneven cobblestone (CS). Multiple signals were recorded during each trial, and signal labels and descriptions can be found in Table 1. As an example, Figure 2 illustrates the magnetic field signal pattern recorded from the right shank sensor placed on Participant 10 while walking on each surface type.

Participants were instructed to perform their walking trials as naturally as possible, which involved walking at their usual pace and allowing their arms to swing freely. Trials conducted as straight walking had an average duration of 16.4 ± 4.2 s. Regarding the organization of the walking conditions, participants executed their trials in a random sequence and had intervals for rest between trials. Each participant completed a total of six trials for each walking condition, resulting in a grand total of 30 participants×9 surface×6 trials=1260 trials (excluding calibration). During walking, a researcher accompanied the participant with a recording device to enhance signal quality. Data recording was carried out using the MTw Awinda software from Xsens, located in Enschede, The Netherlands, at a sampling frequency of 100 Hz. The recorded data encompassed gait information along with time-related details, all managed by the software.

### 2.2. Preprocessing

The preprocessing steps applied to the signals are outlined below:

1. Smoothing: the raw gait data underwent smoothing using a second-order low-pass Butterworth filter with a cut-off frequency of 6 Hz [18];
2. Cleaning: Missing values were addressed by filling them with preceding values if available; otherwise, they were replaced with subsequent values. Specifically, all values collected from the left thigh sensor during 14 trials were missing, leading to their complete removal;
3. Data Combination: data from different sensors and various signals were aggregated to generate inputs for the CNN;
4. Segmentation: segmentation was performed using non-overlapping sliding windows of varying lengths [14,16];
5. Standardization: The gait data were rescaled to fit within the [0, 1] range, utilizing values from the training set of each fold. This process was undertaken to improve performance and accelerate training [19].

### 2.3. Input Generation for the CNN

To maintain computational efficiency and practicality in our approach, we deliberately chose to use 2D matrices as inputs for the 1D-CNN. This decision stems from several key considerations. Firstly, our dataset primarily comprises time-series signals collected from wearable sensors. These signals represent essential information about an individual’s gait and motion, which are vital for FRA. Instead of employing complex and computationally expensive signal transformation techniques, such as time-frequency representations (e.g., spectrograms or scalograms), we opted to work directly with the raw signal data. This choice not only streamlines our computational process but also ensures that we retain the original temporal characteristics of the signals.

Furthermore, employing time-frequency representations would significantly impact the inference speed of our FRA system. Given the real-time nature of fall risk assessment in practical scenarios, maintaining computational efficiency is crucial for timely and reliable predictions. By using 2D matrices representing the raw signals, we can process the data efficiently without compromising the system’s responsiveness.

Additionally, our approach places a strong emphasis on signal fusion, wherein we combine information from multiple sensors and signal groups to improve classification accuracy. Using 2D matrices as inputs allows us to seamlessly integrate this signal fusion step into our framework. It facilitates the combination of signals from different sensors and locations, enabling us to identify the most relevant signals, the most related sensor locations, and the optimal window length for FRA. To achieve these objectives, we designed the following three sets of experiments using data from all participants. In the initial set of experiments, we scrutinized various signals and signal combinations to identify the most effective ones. The second set of experiments was dedicated to investigating sensor configurations, aiming to identify the optimal arrangement. Lastly, in the third set of experiments, we delved into the analysis of window lengths to determine the most suitable duration for achieving our research goals.

Experiment Set 1: To identify the optimal signal combination, various signals and their combinations were examined using an experimental procedure similar to a sequential feature selection algorithm [20,21]. The study conducted by Hu et al. [16] used a segment size of 400 samples, and they attained the highest accuracy with signals recorded from shank sensors. Consequently, input matrices for this set of experiments consisted of stacked 400-sample-long segments from various signals acquired from shank sensors. After smoothing, cleaning, and segmentation, the experiments were conducted as follows:

(1) Select a signal group for input generation;
(2) Form input matrices by stacking preprocessed signals from the selected group. For example, signal group Acc contains Acc_X, Acc_Y, and Acc_Z, and stacking those signals collected from shank sensors generates matrices with a size of 400 × 6;
(3) Calculate the initial cross-validated accuracy;
(4) Repeat step 1 through step 3 for all signal groups;
(5) Select the signal group with the highest initial cross-validated accuracy;
(6) Generate input matrices by stacking the previously selected signal groups and one of the remaining signal groups;
(7) Calculate the cross-validated accuracy using the combined input matrices;
(8) If the accuracy improves, keep the current signal group in the selected set; otherwise, discard it;
(9) Repeat steps 6, 7, and 8 for all remaining signal groups until accuracy stops improving.

Experiment Set 2: Different sensor locations and combinations were employed to determine the best-performing sensor configuration. To manage the experiment load, not all possible combinations were tested. The optimal signal combination established in the first set of experiments was utilized in these trials. After applying smoothing, cleaning, and segmentation, the experiments proceeded as outlined below:

(1) Select a sensor for input generation;
(2) Create input matrices for cross-validation by stacking preprocessed signals from the selected sensor (only using the signals determined as optimal in the first set of experiments);
(3) Calculate cross-validated accuracy;
(4) Repeat steps 1 to 3 for all sensors;
(5) Arrange sensors in descending order based on their cross-validated accuracy;
(6) Generate input matrices by stacking signals from the top two sensors;
(7) Calculate cross-validated accuracy;
(8) If cross-validated accuracy does not improve, exclude signals from the second sensor;
(9) Generate input matrices by stacking matrices created in previous steps and signals from the next sensor;
(10) Calculate cross-validated accuracy;
(11) Exclude signals from the sensor used in step 9 if cross-validated accuracy does not improve;
(12) Repeat steps 9 to 11 for all sensors.

Experiment Set 3: While previous studies adopted two different segmentation approaches, namely, window-based segmentation [14,16,22] and gait cycle-based segmentation [15,17], we chose to use the former approach. This decision was made because gait cycle-based segmentation can introduce challenges related to reliable gait cycle detection, especially in diverse environments [23]. Given that previous studies employed windows with varying lengths, we conducted experiments using different window lengths to determine the most suitable one. The best-performing signal combination determined in set 1 and sensor configuration determined in set 2 were used with the following window lengths to segment signals: 100 samples, 200 samples, 300 samples, 400 samples, and 500 samples. The number of samples for each class with respect to window length were listed in Table 2.

### 2.4. Model Architecture

Recently, deep learning, particularly CNN, has gained significant popularity among researchers due to its state-of-the-art performance and wide-ranging applications across various research domains, including computer vision [24,25,26], image processing [27,28,29], biomedical image and signal classification [10,30,31,32,33,34,35], and authentication systems [36,37]. CNNs are feed-forward neural networks inspired by the human visual cortex, designed to directly extract features from input data. A typical CNN model comprises several concatenated layers, including convolution, activation, pooling layers, and a dense layer with softmax activation [38]. In this context, we have developed a simple model consisting of several blocks, each containing convolution, activation, batch normalization, and pooling layers. The main aim is to achieve high accuracy and fast training and inference speed. The convolution layers play a key role in generating feature maps within the model, starting from low-level features such as pixel groups and edges and progressing to high-level patterns [39]. Each block in our model begins with a convolution layer. To introduce non-linearity into the model, a non-linear activation layer follows each convolution layer. We opted for the ReLU layer, known for addressing vanishing or exploding gradient issues and widely used in deep learning [40]. A batch normalization layer was applied after each ReLU layer to normalize activations in intermediate layers, expedite training, and enhance model performance [41]. Subsequently, each batch normalization layer was followed by a max-pooling layer, a commonly used pooling layer that reduces the dimension of the pooling layer and imparts invariance to minor variations [42]. To mitigate overfitting, we incorporated three dropout layers after the last three max-pooling layers (layers 12, 17, and 22). These dropout layers randomly set input units to zero at a specified frequency [43]. Finally, a flatten layer was employed to convert the feature matrix into a vector before classification by a dense layer with softmax activation. The model was implemented using Keras with the TensorFlow backend.

Details of the implemented model are listed in Table 3. The model architecture was established using our prior knowledge and initial experimentation. In these preliminary experiments, we assessed the model’s performance during training to determine the appropriate number of blocks and filters. We adjusted the model’s capacity, which included the number of blocks and filters, based on the training progress. This iterative process allowed us to create a streamlined structure that achieved a balance between fast training and inference speed while maintaining high accuracy. Significantly, the memory space occupied by our model varied, ranging from 357.79 KB to 1.92 MB, depending on the inputs.

### 2.5. Model Training

The model underwent training for 100 epochs employing a sparse categorical cross-entropy loss function and the Adam optimizer [44] with a learning rate set to 0.001 and a batch size of 32. To prevent overfitting, early stopping was implemented. This mechanism halts training when validation accuracy or loss ceases to improve. The early stopping criteria were defined as follows: if the validation accuracy showed no improvement for 25 epochs, the training process was terminated, and the model with the highest validation accuracy was selected for prediction. Additionally, in case the validation accuracy did not increase over a span of 10 epochs, the learning rate was reduced by a factor of 0.1, effectively reducing the model’s generalization error. The training process was performed on a free instance of Google Colaboratory with a T4 GPU.

To assess the CNN model’s ability to generalize for the detection of walking surface conditions, we conducted a stratified 6-fold cross-validation. The data were partitioned by trials, ensuring that class ratios were maintained across the training, validation, and test sets. Specifically, the dataset was divided into six subsets, with each subset containing one trial of all subjects and surface types. Four subsets were allocated for training, one subset for validation, and the remaining one for testing. This procedure was repeated six times, cycling through each subset for testing. Performance metrics were computed based on the aggregated predictions from each fold.

### 2.6. Performance Metrics

The performance of each experiment was evaluated based on the aggregated predictions from the 6-fold cross-validation using metrics such as accuracy, weighted precision, recall, and F1-score [45]. Per-class precision ($PR_{i}$), recall ($RE_{i}$), and F1-score ($F1_{i}$) are calculated as follows:

(1) $$PR_{i}=\frac{e_{ii}}{\sum_{j=1}^{N_{c}}e_{ij}}\;$$

(2) $$RE_{i}=\frac{e_{ii}}{\sum_{j=1}^{N_{c}}e_{ji}}$$

(3) $$F1_{i}=\frac{2\times PR_{i}\times RE_{i}}{PR_{i}+RE_{i}}$$

where $e_{ij}$ represents the element at the *i*th row and *j*th column of the confusion matrix, and $N_{c}$ stands for the number of classes. The weighted average of a metric is calculated as the average of the values of that metric for each class, weighted by the number of samples in each class. Lastly, accuracy (*ACC*) is defined as:

(4) $$ACC=\frac{\sum_{i=1}^{N_{c}}e_{ii}}{\sum_{i=1}^{N_{c}}\sum_{j=1}^{N_{c}}e_{ij}}$$

## 3. Results

Several experiments were conducted to find the best signal combination, sensor placement, and window length (L). Even though there were 7 signal groups×6 sensors×5 window lengths=210 possible combinations just in the case of using single sensors and signal groups, procedures explained in Section 2.3 were followed to keep the number of experiments limited. Therefore, three experimental sets were formed. Each set was used for different purposes. The first set was used to find the best signal or combination of signals. The second set was employed to determine the best sensor configuration. The last set was exploited to select the optimal window length.

Twenty-seven experiments were conducted within the scope of the first set. Left and right shank sensors and 400 sample points for each segment were used throughout the experiments. While these parameters were kept constant, different signal groups were tried using a procedure similar to the sequential feature selection algorithm. As a result, Mag, FreeAcc, YPR, Acc, VelInc were determined as the best signal combination, and they achieved an accuracy of 0.961. Performance results for all experiments are listed in Table 4. Mag obtained the best, and Ori obtained the worst accuracy in the individual signal group. Even though fusing Mag and FreeAcc increased accuracy by 3.5% (from 0.923 to 0.955), fusing the others slightly improved accuracy by just 0.6% (from 0.955 to 0.961). Furthermore, the fusing of Gyr and Ori almost did not affect accuracy. Results showed that individual signal selection could dramatically affect the system’s overall performance (accuracy from 0.629 to 0.923), and signal fusion could improve it further (accuracy from 0.923 to 0.961).

**Figure 3:**
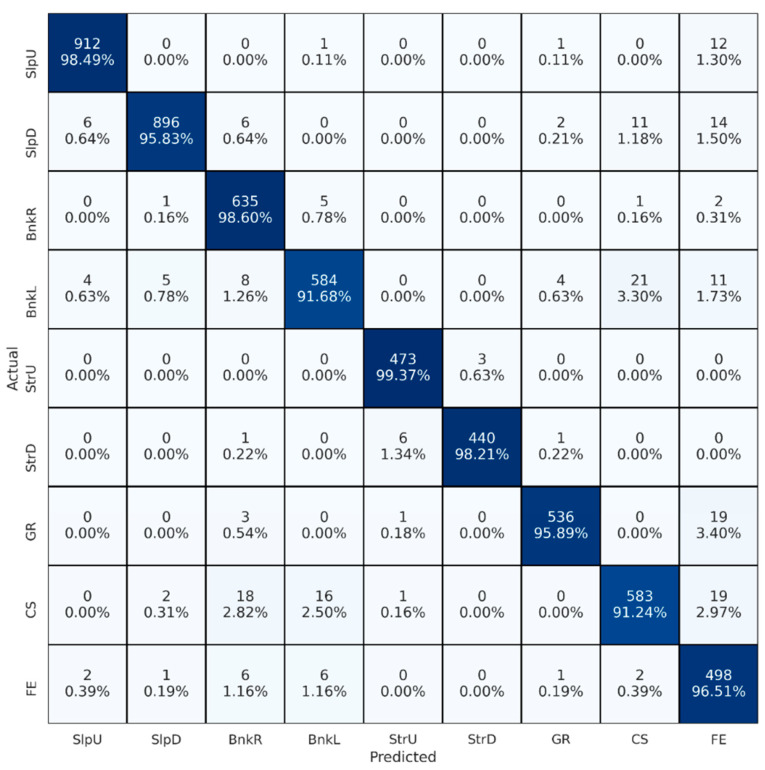
Confusion matrix for the best-performing signal combination. BnkR and BnkL indicate surfaces banked to the right and left, SlpD and SlpU represent surfaces with downhill and uphill slopes, and StrD and StrU indicate descending and ascending stairs. CS, FE, and GR stand for cobblestone, flat even, and grass surfaces, respectively. Color intensity reflects the percentage of correct predictions for each walking surface condition, with darker shades indicating higher values.

Figure 3 depicts the confusion matrix obtained for the best setting of the first set of experiments. Rows and columns represent actual and predicted classes, respectively. Diagonal cells show correctly classified segments, while non-diagonal cells indicate misclassified segments. Percentage values are the counts normalized by the total number of actual classes. Therefore, percentage values in diagonal cells are the recall of each class. In terms of classes, upstairs (StrU) showed the highest recall (0.993), and uneven cobblestone (CS) showed the lowest (0.912).

**Figure 4:**
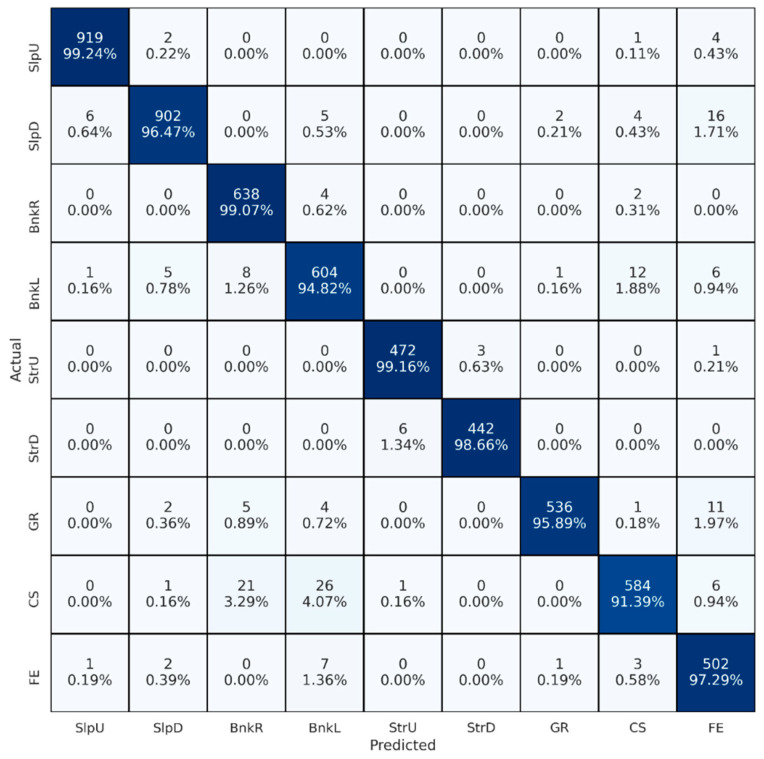
Confusion matrix for the best-performing sensor placement. BnkR and BnkL indicate surfaces banked to the right and left, SlpD and SlpU represent surfaces with downhill and uphill slopes, and StrD and StrU indicate descending and ascending stairs. CS, FE, and GR stand for cobblestone, flat even, and grass surfaces, respectively. Color intensity reflects the percentage of correct predictions for each walking surface condition, with darker shades indicating higher values.

In the second experimental set, we conducted twelve experiments. The initial step involved determining the best signal combination, which was established based on the outcomes of the first set of experiments. Throughout these experiments, we consistently employed 400-sample-long segments. Our systematic approach began with the selection of individual sensors, from which we generated input matrices using preprocessed signals. We considered the performance of each sensor from previous experiments, calculating cross-validated accuracy and arranging them in order of performance. Subsequently, we combined signals from the top two sensors to evaluate accuracy. If no improvements were observed, we excluded signals from the second sensor, continuing this iterative process by combining signals from previous steps with the next sensor and reassessing accuracy. Sensors that did not contribute to improved accuracy were progressively excluded from consideration, ultimately leading us to determine the best-performing sensor combination. Performance results for all twelve experiments are listed in Table 5. In the case of single sensors, left and right shank sensors achieved very similar performance (0.935 accuracy). While left thigh, right thigh, and trunk sensors showed similar results, wrist sensors reported significantly lower performance (0.760 accuracy). In the case of multiple sensors, the left shank, right shank, trunk, and right thigh sensors demonstrated the best performance (0.969 accuracy). However, using just left and right shank sensors showed very similar results (0.961 accuracy), and even using a single sensor achieved comparably accurate performance (0.935 accuracy). Combining left thigh and wrist sensors with the rest had little or no effect on performance. Therefore, we concluded that left and right shank sensors could be used instead of the best sensor configuration, which requires four sensors for user convenience. Finally, the confusion matrix obtained for the best setting of the second set of experiments, which conveys similar class-based results to the previous one, is presented in Figure 4.

**Figure 5:**
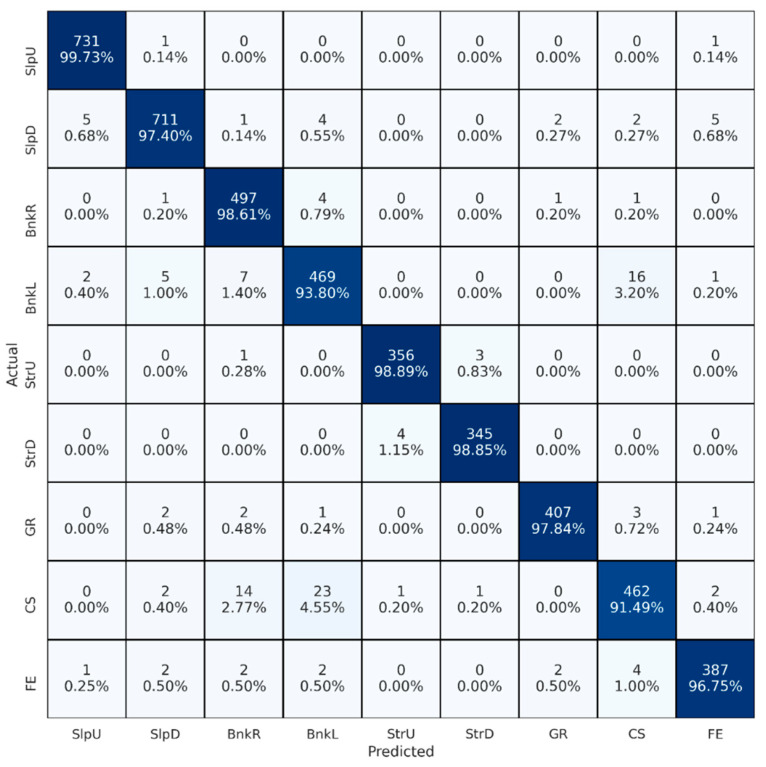
Confusion matrix for the best-performing window size. BnkR and BnkL indicate surfaces banked to the right and left, SlpD and SlpU represent surfaces with downhill and uphill slopes, and StrD and StrU indicate descending and ascending stairs. CS, FE, and GR stand for cobblestone, flat even, and grass surfaces, respectively. Color intensity reflects the percentage of correct predictions for each walking surface condition, with darker shades indicating higher values.

Building on the findings from the first and second experimental sets, which had already established the best signal combination and sensor configuration, we explored various window lengths (100, 200, 300, 400, and 500 samples) for segmenting continuous signals. The results of these experiments, presented in Table 6, revealed that using a 500-sample window yielded the highest performance with an accuracy of 0.971, while the shortest window led to the lowest performance with an accuracy of 0.958. Interestingly, the results indicated that overall performance improved as the window length increased, despite generating fewer segments (i.e., reducing the number of samples) with longer windows. Additionally, we included the confusion matrix for the best-performing case, as illustrated in Figure 5.

## 4. Discussion

This study aimed to classify irregular walking conditions using IMU-based gait time series and CNN. Since different settings were possible in terms of signal groups, sensor configuration, and window length, extensive analysis was carried out using three sets of experiments. These experiments determined the most relevant signal(s), the best sensor configuration, and the optimal window length. The results of the first set of experiments, as shown in Table 4, revealed the capability to classify nine distinct walking conditions with a satisfactory accuracy of 0.923 using only 3D magnetometer signals. This finding could benefit real-time applications, especially those with limited memory and computing power. Furthermore, relying on a single signal and, as a result, one measurement unit (magnetometer) could enhance the power efficiency of wearable devices that measure, record, and process human gait. Finally, the results indicated that accuracy can be improved by 3.5% when fusing only two signal groups. This presents an optimal configuration that enhances performance while minimizing the energy consumption of wearable devices. Additionally, the memory space occupied by the model decreased significantly from 1.39 MB to 560.29 KB for a window length of 400 samples.

**Figure 6:**
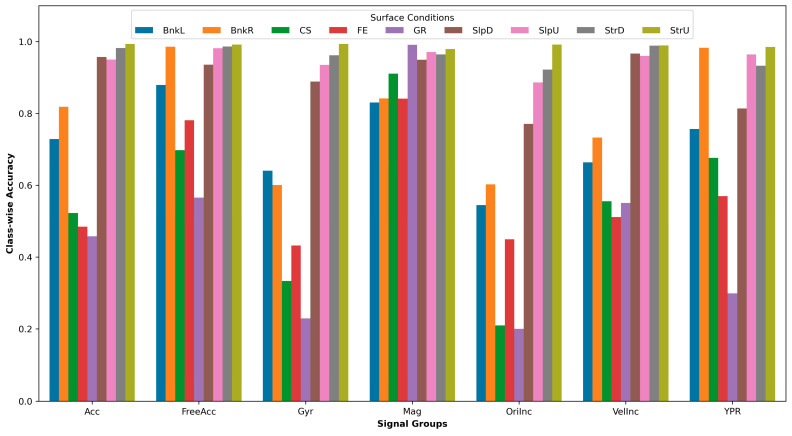
Class-wise accuracies for each surface condition and signal group. BnkR and BnkL indicate surfaces banked to the right and left, SlpD and SlpU represent surfaces with downhill and uphill slopes, and StrD and StrU indicate descending and ascending stairs. CS, FE, and GR stand for cobblestone, flat even, and grass surfaces, respectively. Acc, FreeAcc, Gyr, Mag, VelInc, and OriInc stand for acceleration, gravity-subtracted acceleration, gyroscope, magnetic field, velocity increment, and orientation increment quaternion, respectively. YPR represents yaw, pitch and roll signals.

Figure 6 shows a bar plot depicting class-wise accuracies for each surface condition. The data are based on sensors located on the shanks, using a 400-sample window. Notably, all signal types consistently demonstrated the ability to accurately detect StrU, StrD, SlpU, and SlpD conditions. This suggests that these surface conditions have distinct gait patterns and properties that can be effectively captured by various signal types. However, accurately detecting GR, CS, and FE proved to be the most difficult. Additionally, Mag signals consistently performed better than other signals on most surface conditions, possibly because of the sensors’ orientation and placement on the lower extremity, which facilitates the capturing of significant magnetic field variations associated with surface differences. On the other hand, Gyr and OriInc, a signal group derived from Gyr, exhibited the poorest performance, possibly due to their primary focus on rotational movements instead of translational shifts during walking. This restriction impeded their capacity in distinguishing between various surface conditions, especially GR, CS, and FE.

Sensor placement was investigated within the second set of experiments. The results, as shown in Table 5, revealed that sensors closer to walking surfaces mainly demonstrated better performance than those far from the walking surfaces, especially wrist sensors. Three reasons can justify this: the more direct effects of irregularities in walking surfaces on the lower extremity; gait adaptations of the lower extremity that aim to keep the center of gravity of the human body and upper body relatively stable; and inconsistency in the involuntary movement of the upper extremity despite its role in body balance. Additionally, the number of sensors and their placement are essential factors affecting accuracy, hardware requirements, power consumption, inference speed, and user convenience. The results showed that using four sensors achieved the highest accuracy of 0.969. However, using four sensors (left and right shanks, trunk, and right thigh) may cause discomfort to users. User comfort could be improved by using just two sensors (left and right shank sensors) while sacrificing a small portion of accuracy (from 0.969 to 0.961).

Segmenting continuous signals is necessary for many classification tasks because: (1) most classifiers, such as CNN, accept fixed-size inputs; (2) continuous signals are hard to process; and (3) continuous signals cannot be used for real-time applications. Thus, segmentation was a necessary step for the proposed framework. Moreover, segment length affects performance since it determines how much valuable information a segment contains. Therefore, we analyzed segment length in the third set of experiments. The results, as shown in Table 6, reported that the longest segment size (500 samples) achieved the highest performance. However, longer segments would increase detection time for real-time applications. To classify 500-sample-long segments, sensors measure gait for five seconds and save it to the memory. Then, the segment is processed (filtered and cleaned) and classified by a trained CNN model. As a result, the total prediction time of a single segment equals the sum of the times taken at each stage of this three-stage process. Delayed surface detection could be problematic for the reliability of FRA methods. The results demonstrated that total prediction time could be at least five times lower by giving up just 1.3% of the highest accuracy and using 100 samples instead of 500 samples.

Table 7 summarizes studies on gait-based automated detection of walking surface conditions, including the number of participants and surfaces, features and models used for classification, signal types, sensor locations, segmentation types, and performance results for each study. Despite the high accuracy obtained in the study conducted by Hu et al. [14], they employed data from 17 participants who performed walking trials on just two different surfaces (flat and uneven surfaces). Hashmi et al. [15] worked with a dataset containing gait data collected from 40 participants while walking on six different surfaces. They relied on stride detection for the segmentation of continuous gait signals. Even though this type of segmentation preserves gait-related information, the overall accuracy of the system greatly depends on the success of the stride detection algorithm used. These algorithms may not be reliable in real-world conditions. In fact, the same unreliability is applied to FRAs, which is the main reason for the proposal of environment-aware FRAs in the first place. The other studies, including ours, employed the same dataset. However, Hu et al. [16] used data for seven surfaces instead of nine. They investigated the effects of sensor locations by using sensors individually and all the sensors together. Similar analyses were conducted by Shah et al. [17] and McQuire [22] as well. However, their studies lack a systematic analysis of sensor locations and the gait signals utilized. Furthermore, none of the previous studies analyzed and discussed the segment length in terms of detection accuracy and speed. We proposed a gait-based automated surface detection method while systematically analyzing sensor location, signals, and segment length. Our method avoided the use of LSTM, which is slower than CNN, and gait cycle-based segmentation techniques, which may lead to lower reliability for the overall system performance. There are several limitations to this study that should be mentioned. Weather conditions that change the slipperiness of walking surfaces were not covered, and even though the study employed data for nine surfaces, there are many more surface conditions that affect individuals’ gait patterns. Additionally, static obstacles that could also affect gait patterns were not taken into account. Therefore, future research should consider the effects of static obstacles on walking areas and include different weather conditions and a greater variety of surface conditions to provide a more comprehensive understanding of gait patterns in different environments.

In order to develop more effective environment-aware free-living FRAs, it is crucial for researchers to improve the accuracy and comprehensiveness of the information on the properties of different walking environments. This can be achieved through the automated identification of environmental factors that contribute to falls and missteps, such as static obstacles and uneven and slippery surfaces. Such information can then be integrated into IMU-based environment-aware free-living FRA methods, which would facilitate the development of more specific intervention strategies for reducing falls and improving balance in older adults. For instance, by identifying a high frequency of slips while walking on indoor tiles, non-slip flooring materials could be installed to prevent future imbalance events.

## 5. Conclusions

In conclusion, our research harnessed the capabilities of a convolutional neural network model to effectively classify various irregular walking conditions. We conducted a systematic analysis leveraging an open-access database featuring gait signals collected from participants wearing IMU sensors while navigating nine distinct outdoor surfaces. This analysis explored the impact of sensor placement, gait signal fusion, and signal segmentation on our classification performance.

Our research findings suggest the feasibility of attaining satisfactory performance even when employing a restricted set of signals and sensors. Furthermore, we have substantiated that achieving an equilibrium between resource utilization and accuracy can be effectively realized through judicious sensor placement, the implementation of advanced signal fusion techniques, and the meticulous selection of optimal segment sizes. This underscores the potential for practical and resource-efficient implementations in real-world applications.

The significance of our work extends beyond the confines of this study. We anticipate that our results and analysis will serve as a crucial stepping stone towards the development of more reliable fall risk assessments (FRAs), particularly those capable of adapting to dynamic environments. This research lays the foundation for the creation of environment-aware free-living FRAs, with the potential to improve safety and quality of life for individuals at risk of falls. We anticipate that our results and analysis will contribute to the ongoing efforts in fall risk assessment and healthcare.

## Figures and Tables

**Table 1:** Signal labels and descriptions. Acc, FreeAcc, Gyr, Mag, VelInc, and OriInc stand for acceleration, gravity-subtracted acceleration, gyroscope, magnetic field, velocity increment, and orientation increment quaternion, respectively. YPR represents yaw, pitch and roll signals. X, Y, and Z denote the axes. q0, q1, q2, and q3 represent individual quaternions.

Signal Label	Description
Acc: Acc_X, Acc_Y, Acc_Z	3D acceleration
FreeAcc: FreeAcc_X, FreeAcc_Y, FreeAcc_Z	3D acceleration (gravity subtracted)
Gyr: Gyr_X, Gyr_Y, Gyr_Z	3D rate of turn
Mag: Mag_X, Mag_Y, Mag_Z	3D magnetic field
VelInc: VelInc_X, VelInc_Y, VelInc_Z	3D velocity increment
Ori: OriInc_q0, OriInc_q1, OriInc_q2, OriInc_q3	Orientation increment quaternion
YPR: Yaw, Pitch, Roll	3D Euler angles

**Table 2:** The number of samples for each class with respect to window length. BnkR and BnkL indicate surfaces banked to the right and left, SlpD and SlpU represent surfaces with downhill and uphill slopes, and StrD and StrU indicate descending and ascending stairs. CS, FE, and GR stand for cobblestone, flat even, and grass surfaces, respectively.

Window Length	SlpU	SlpD	BnkR	BnkL	StrU	StrD	GR	CS	FE	Total
100	3994	4011	2843	2800	2150	2042	2526	2816	2354	25,636
200	1950	1961	1374	1356	1031	985	1227	1364	1131	12,579
300	1265	1276	889	869	658	626	775	877	723	8258
400	926	935	644	637	476	448	559	639	516	6180
500	733	730	504	500	360	349	416	505	400	4997

**Table 3:** Details of the parameters for each layer in the proposed 1D-CNN model.

Block No	Layer No	Layer	Parameters
1	1	Convolution1D	filters = 64, kernel_size = 3, strides = 1
	2	ReLU	-
	3	Batch Norm.	-
	4	Max Pooling1D	pool_size = 3, strides = 3
2	5	Convolution1D	filters = 64, kernel_size = 3, strides = 1
	6	ReLU	-
	7	Batch Norm.	-
	8	Max Pooling1D	pool_size = 3, strides = 3
3	9	Convolution1D	filters = 128, kernel_size = 3, strides = 1
	10	ReLU	-
	11	Batch Norm.	-
	12	Max Pooling1D	pool_size = 3, strides = 3
	13	Dropout	rate = 0.5
4	14	Convolution1D	filters = 128, kernel_size = 3, strides = 1
	15	ReLU	-
	16	Batch Norm.	-
	17	Max Pooling1D	pool_size = 3, strides = 3
	18	Dropout	rate = 0.5
5	19	Convolution1D	filters = 128, kernel_size = 3, strides = 1
	20	ReLU	-
	21	Batch Norm.	-
	22	Max Pooling1D	pool_size = 3, strides = 3
	23	Dropout	rate = 0.5
6	24	Flatten	-
	25	Dense	units = 9, activation = ’softmax’

**Table 4:** Performance results for the first set of experiments. Acc, FreeAcc, Gyr, Mag, VelInc, and OriInc stand for acceleration, gravity-subtracted acceleration, gyroscope, magnetic field, velocity increment, and orientation increment quaternion, respectively. ShankR and ShankL refer to the right and left shank, respectively. The superscript * indicates that the accuracy for signal groups including “Mag, FreeAcc, YPR” was slightly more significant than that for signal groups including “Mag, FreeAcc”.

Signal Group(s)	Sensor Locations	L	PR	RE	F1	ACC
Mag	ShankL, ShankR	400	0.925	0.923	0.923	0.923
FreeAcc	ShankL, ShankR	400	0.872	0.869	0.865	0.869
YPR	ShankL, ShankR	400	0.807	0.810	0.804	0.810
Acc	ShankL, ShankR	400	0.808	0.803	0.800	0.803
VelInc	ShankL, ShankR	400	0.783	0.784	0.781	0.784
Gyr	ShankL, ShankR	400	0.699	0.694	0.680	0.694
Ori	ShankL, ShankR	400	0.632	0.629	0.608	0.629
Mag, FreeAcc	ShankL, ShankR	400	0.956	0.955	0.955	0.955
Mag, Acc	ShankL, ShankR	400	0.948	0.947	0.947	0.947
Mag, YPR	ShankL, ShankR	400	0.944	0.944	0.943	0.944
Mag, VelInc	ShankL, ShankR	400	0.944	0.943	0.943	0.943
Mag, Ori	ShankL, ShankR	400	0.932	0.930	0.930	0.930
Mag, Gyr	ShankL, ShankR	400	0.926	0.923	0.924	0.923
Mag, FreeAcc, YPR *	ShankL, ShankR	400	0.956	0.955	0.955	0.955
Mag, FreeAcc, Acc	ShankL, ShankR	400	0.955	0.954	0.954	0.954
Mag, FreeAcc, Ori	ShankL, ShankR	400	0.953	0.952	0.952	0.952
Mag, FreeAcc, Gyr	ShankL, ShankR	400	0.952	0.951	0.951	0.951
Mag, FreeAcc, VelInc	ShankL, ShankR	400	0.951	0.950	0.950	0.950
Mag, FreeAcc, YPR, Acc	ShankL, ShankR	400	0.957	0.956	0.956	0.956
Mag, FreeAcc, YPR, VelInc	ShankL, ShankR	400	0.954	0.953	0.953	0.953
Mag, FreeAcc, YPR, Ori	ShankL, ShankR	400	0.954	0.953	0.953	0.953
Mag, FreeAcc, YPR, Gyr	ShankL, ShankR	400	0.952	0.951	0.951	0.951
Mag, FreeAcc, YPR, Acc, VelInc	ShankL, ShankR	400	0.963	0.961	0.962	0.961
Mag, FreeAcc, YPR, Acc, Ori	ShankL, ShankR	400	0.959	0.958	0.958	0.958
Mag, FreeAcc, YPR, Acc, Gyr	ShankL, ShankR	400	0.946	0.956	0.955	0.955
Mag, FreeAcc, YPR, Acc, VelInc, Gyr	ShankL, ShankR	400	0.961	0.960	0.960	0.960
Mag, FreeAcc, YPR, Acc, VelInc, Ori	ShankL, ShankR	400	0.960	0.960	0.960	0.960

**Table 5:** Performance results for the second set of experiments. Acc, FreeAcc, Mag, and VelInc stand for acceleration, gravity-subtracted acceleration, magnetic field, and velocity increment, respectively. YPR denotes yaw, pitch, and roll. ShankR and ShankL refer to the right and left shank, respectively, while ThighR and ThighL denote the right and left thigh.

Signal Groups	Sensor Location(s)	L	PR	RE	F1	ACC
Mag, FreeAcc, YPR, Acc, VelInc	ShankL	400	0.939	0.935	0.935	0.935
Mag, FreeAcc, YPR, Acc, VelInc	ShankR	400	0.937	0.935	0.936	0.935
Mag, FreeAcc, YPR, Acc, VelInc	ThighL	400	0.872	0.865	0.857	0.865
Mag, FreeAcc, YPR, Acc, VelInc	ThighR	400	0.884	0.881	0.877	0.881
Mag, FreeAcc, YPR, Acc, VelInc	Trunk	400	0.888	0.883	0.874	0.883
Mag, FreeAcc, YPR, Acc, VelInc	Wrist	400	0.764	0.760	0.742	0.760
Mag, FreeAcc, YPR, Acc, VelInc	ShankL, ShankR	400	0.963	0.961	0.962	0.961
Mag, FreeAcc, YPR, Acc, VelInc	ShankL, ShankR, Trunk	400	0.968	0.968	0.968	0.968
Mag, FreeAcc, YPR, Acc, VelInc	ShankL, ShankR, Trunk, ThighR	400	0.969	0.969	0.969	0.969
Mag, FreeAcc, YPR, Acc, VelInc	ShankL, ShankR, Trunk, ThighR, ThighL	400	0.966	0.965	0.965	0.965
Mag, FreeAcc, YPR, Acc, VelInc	ShankL, ShankR, Trunk, ThighR, Wrist	400	0.969	0.969	0.969	0.969
Mag, FreeAcc, YPR, Acc, VelInc	ShankL, ShankR, Trunk, ThighR, Wrist, ThighL	400	0.967	0.966	0.966	0.966

**Table 6:** Performance results for the third set of experiments. Acc, FreeAcc, Mag, and VelInc stand for acceleration, gravity-subtracted acceleration, magnetic field, and velocity increment, respectively. YPR denotes yaw, pitch, and roll. ShankR and ShankL refer to the right and left shank, respectively, while ThighR and ThighL denote the right and left thigh.

Signal Groups	Sensor Locations	L	PR	RE	F1	ACC
Mag, FreeAcc, YPR, Acc, VelInc	ShankL, ShankR, Trunk, ThighR	100	0.958	0.958	0.958	0.958
Mag, FreeAcc, YPR, Acc, VelInc	ShankL, ShankR, Trunk, ThighR	200	0.967	0.967	0.967	0.967
Mag, FreeAcc, YPR, Acc, VelInc	ShankL, ShankR, Trunk, ThighR	300	0.967	0.967	0.967	0.967
Mag, FreeAcc, YPR, Acc, VelInc	ShankL, ShankR, Trunk, ThighR	400	0.969	0.969	0.969	0.969
Mag, FreeAcc, YPR, Acc, VelInc	ShankL, ShankR, Trunk, ThighR	500	0.971	0.971	0.971	0.971

**Table 7:** A summary of studies on gait-based walking surface condition detection. kNN: k-nearest neighbors, NB: naïve Bayes, QDA: quadratic discriminant analysis, DNN: deep neural network.

Study	Number of Participants	Number of Surfaces	Signals	Sensor Location	Segmentation	Features	Classifier	Accuracy (%)
Ref. [14]	17	2	Acc, Gyr, Mag	Lower back	4 s window	Learned	LSTM	96.3
Ref. [15]	40	6	Acc, Gyr	Chest and lower back	Stride-based	Tempo-spectral	RF, SVM	89.0
Ref. [16]	30	7	Acc, Gyr	Shanks, thighs, lower back, wrist, all combined	4 s window	Learned	CNN, LSTM	92.0
Ref. [17]	30	9	Acc, Gyr, Mag	Shanks, thighs, lower back, wrist, combination of lower sensors, all combined	Gait cycle-based	Linear discriminant analysis-based	Multi-layer perceptron	97.0
Ref. [22]	30	9	Acc, Gyr, Mag	Shanks, thighs, lower back, wrist	2.56 s window	Time and frequency	SVM, RF, kNN, NB, QDA, DNN	94.3
This Study	30	9	Acc, Gyr, Mag, FreeAcc, VelInc, Ori, YPR, combinations of these	Shanks, thighs, lower back, wrist, combinations of these	1, 2, 3, 4, and 5 s windows	Learned	CNN	97.1

## Data Availability

The data used in this study are openly available in FigShare at https://doi.org/10.6084/m9.figshare.c.4892463.v1 (accessed on 21 March 2022), reference number [18].
